# Supplementary material for: Auditory cortical micro-networks show differential connectivity during voice and speech processing in humans
Source: Commun Biol. 2021 Jun 25;4:801. doi: 10.1038/s42003-021-02328-2 (PMC8233416; doi:10.1038/s42003-021-02328-2)
Supplement: Supplementary file 2 — Reporting Summary [file 42003_2021_2328_MOESM2_ESM.pdf]

nature research

Corresponding author(s): Sascha Frühholz

Last updated by author(s): Jun 2, 2021

Reporting Summary

Nature Research wishes to improve the reproducibility of the work that we publish. This form provides structure for consistency and transparency in reporting. For further information on Nature Research policies, see our Editorial Policies and the Editorial Policy Checklist.

Statistics

For all statistical analyses, confirm that the following items are present in the figure legend, table legend, main text, or Methods section.

n/a	Confirmed	
☐	☒	The exact sample size (*n*) for each experimental group/condition, given as a discrete number and unit of measurement
☐	☒	A statement on whether measurements were taken from distinct samples or whether the same sample was measured repeatedly
☐	☒	The statistical test(s) used AND whether they are one- or two-sided<br>*Only common tests should be described solely by name; describe more complex techniques in the Methods section.*
☒	☐	A description of all covariates tested
☐	☒	A description of any assumptions or corrections, such as tests of normality and adjustment for multiple comparisons
☐	☒	A full description of the statistical parameters including central tendency (e.g. means) or other basic estimates (e.g. regression coefficient) AND variation (e.g. standard deviation) or associated estimates of uncertainty (e.g. confidence intervals)
☒	☐	For null hypothesis testing, the test statistic (e.g. *F*, *t*, *r*) with confidence intervals, effect sizes, degrees of freedom and *P* value noted<br>*Give P values as exact values whenever suitable.*
☐	☒	For Bayesian analysis, information on the choice of priors and Markov chain Monte Carlo settings
☐	☒	For hierarchical and complex designs, identification of the appropriate level for tests and full reporting of outcomes
☒	☐	Estimates of effect sizes (e.g. Cohen's *d*, Pearson's *r*), indicating how they were calculated

Our web collection on statistics for biologists contains articles on many of the points above.

Software and code

Policy information about availability of computer code

Data collection	Siemens MRI scanner software
Data analysis	Pre-processing<br>- Matlab (version 2018b) with statistical parametric mapping software SPM12 (version 7771, https://www.fil.ion.ucl.ac.uk/spm/software/spm12/)<br>- FieldMap toolbox for SPM12 (version 2.0, https://www.fil.ion.ucl.ac.uk/spm/toolbox/fieldmap/) for calculating static distortions from b0 fieldmap<br>- CAT toolbox for SPM12 (version 12.5, http://www.neuro.uni-jena.de/cat/) for brain segmentation and normalization parameter estimation during pre-processing<br><br>Further analyses<br>- eigenvariate toolbox implemented in SPM12 for summarizing time series data from VOIs associated with the included regressors<br>- DCM12 toolbox (version 7479) implemented in SPM12 for modelling effective functional connectivity<br>- Anatomy toolbox (version 2.2b) for SPM12 to locate regions in the auditory cortex

For manuscripts utilizing custom algorithms or software that are central to the research but not yet described in published literature, software must be made available to editors and reviewers. We strongly encourage code deposition in a community repository (e.g. GitHub). See the Nature Research guidelines for submitting code & software for further information.

Data

Policy information about availability of data

All manuscripts must include a data availability statement. This statement should provide the following information, where applicable:

- Accession codes, unique identifiers, or web links for publicly available datasets
- A list of figures that have associated raw data
- A description of any restrictions on data availability

The data that support the findings of this study are available from the corresponding author upon reasonable request.

Field-specific reporting

Please select the one below that is the best fit for your research. If you are not sure, read the appropriate sections before making your selection.

☐ Life sciences ☒ Behavioural & social sciences ☐ Ecological, evolutionary & environmental sciences

For a reference copy of the document with all sections, see nature.com/documents/nr-reporting-summary-flat.pdf

Behavioural & social sciences study design

All studies must disclose on these points even when the disclosure is negative.

Study description	The study acquired and examined quantitative experimental data.
Research sample	We examined young (i.e., mean age 23.95 years, SD 4.22, range 18-34 y), healthy volunteers (30 males, 22 females). They had normal hearing abilities and normal or corrected-to-normal vision. No participant presented a neurological or psychiatric history.<br>The sample was chosen to be representative for an average population of younger people without impairments. This population was necessary to be able to establish our models under "normal" circumstances.
Sampling strategy	Random sampling procedure. We were able to scan 58 participants, from which we had to exclude 6 because of excessive movement or segmentation failure (see "Data exclusions" below). Thus, the final sample size was N=52.
Data collection	Data collection instruments included an MRI scanner to acquire the fMRI data. Two researchers were present to handle the participants and the MRI scanner.<br>The researchers were not blind to the experimental condition. However, each experimental run contained all experimental conditions and once the experiment started there were no interactions between researchers and participants. The participants were blind to the design, the only thing they knew was that they will have to listen to voices and sounds and press a button once they heard a sound twice in a row
Timing	Data was collected between January 2015 until May 2016.
Data exclusions	Six participants had to be excluded due to excessive movement or unsuccessful T1 segmentation. Both criteria are important for reliable data analysis and thus were pre-defined.
Non-participation	No participants dropped out or declined participation.
Randomization	Participants were not allocated into experimental groups; all participants experienced the same experimental manipulation.

Reporting for specific materials, systems and methods

We require information from authors about some types of materials, experimental systems and methods used in many studies. Here, indicate whether each material, system or method listed is relevant to your study. If you are not sure if a list item applies to your research, read the appropriate section before selecting a response.

Materials & experimental systems

n/a	Involved in the study	
☒	☐	Antibodies
☒	☐	Eukaryotic cell lines
☒	☐	Palaeontology and archaeology
☒	☐	Animals and other organisms
☐	☒	Human research participants
☒	☐	Clinical data
☒	☐	Dual use research of concern

Methods

n/a	Involved in the study	
☒	☐	ChIP-seq
☒	☐	Flow cytometry
☐	☒	MRI-based neuroimaging

Human research participants

Policy information about studies involving human research participants

Population characteristics	See above.
Recruitment	Participants were recruited over the university website and public announcements. An initial pre-screening determined the eligibility of participants in terms of participation requirements (age, MRI-compatibleness, neurological/psychiatric history, vision/hearing condition).
Ethics oversight	Cantonal ethics committee of the Swiss Cantone Geneva.

Note that full information on the approval of the study protocol must also be provided in the manuscript.

Magnetic resonance imaging

Experimental design

Design type	Task; event-related design
Design specifications	Stimuli were recordings of 70 voice sounds (speech, nonverbal/non-speech) and 70 non-voice sounds (animal, natural, and artificial sounds; see Ref. below). Stimuli were presented in an event-related design, with 10% of the sounds repeated randomly for a 1-back task, where participants were asked to press a button upon a consecutive repetition of a sound. Each sound lasted 500ms. All sounds were presented one time in a random order, with a jittered ITI of 4.0-5.5s, and a sound intensity level of 70dB SPL.<br><br>Capilla, A., Belin, P. & Gross, J. The early spatio-temporal correlates and task independence of cerebral voice processing studied with MEG. Cereb. Cortex 23, 1388–1395 (2013).
Behavioral performance measures	Participants' task was to press a button upon a consecutive repetition of a sound (1-back task). The variable recorded was the correct button press. However, no statistics were used to establish correct performance because the task simply served to sustain attention during listening.

Acquisition

Imaging type(s)	functional, structural
Field strength	3 T
Sequence & imaging parameters	Functional: T2*-weighted gradient multiband echo-planar imaging (M-EPI) pulse sequence (acceleration factor 4, 2mm^3 isotropic resolution, 28 slices in a 64x64 matrix, 20% distance factor, TR/TE=650/30ms, flip angle 50°)<br>Structural: 1-mm isotropic resolution, 192 contiguous 1-mm slices, TR/TE/TI=1900/2.27/900ms, FoV 296mm, in-plane resolution of 1x1mm
Area of acquisition	Whole brain acquisition; The brainstem and cerebellum were cut caudally for the sake of complete acquisition of the cerebral cortex.

Diffusion MRI ☐ Used ☒ Not used

Preprocessing

Preprocessing software	Functional imaging time series were pre-processed using SPM12 (version 7771) and the FieldMap (version 2.0) and CAT12 (version 12.5) toolboxes for selected steps. The preprocessing steps included: Combined approach to correct for geometric distortions caused by susceptibility-induced field inhomogeneity and changes in these distortions from head motion (FieldMap), realignment, unwarping, slice time correction (as part of DCM model setup), segmentation (CAT12), co-registration of structural and functional images, normalization to MNI space, spatial smoothing (6-mm FWHM Gaussian kernel).
Normalization	Images were normalized using the normalization parameters estimated in the CAT12 segmentation step. Normalization parameters were estimated using non-linear spatial registration (Dartel). Using those parameters, the anatomical and functional images were spatially normalized to the Montreal Neurological Institute (MNI) stereotactic space.
Normalization template	ICBM152
Noise and artifact removal	Images were corrected for geometric distortions caused by susceptibility-induced field inhomogeneity. A combined approach was used, which corrects for both static distortions and changes in these distortions from head motion. The static distortions were calculated for each subject from a b0 fieldmap that was processed by using the FieldMap toolbox. With these parameters, functional images were then realigned and unwarped, a procedure that allows the measured static distortions to be included in the estimation of distortion changes associated with head motion. Images were smoothed with a 6-mm FWHM Gaussian kernel to ameliorate differences in inter-subject localization and increase the signal-to-noise ratio.
Volume censoring	n/a

Statistical modeling & inference

Model type and settings

Type: mass univariate;
After pre-processing, we estimated the BOLD responses to voices and non-voices using a GLM that contained two regressors for voice sounds (speech, nonverbal) and three regressors for non-voice sounds (animal, natural, and artificial sounds), constructed by convolving a stick function at each sound onset with a canonical hemodynamic response function. Additionally, repeated sounds were modelled in a separate regressor and disregarded in further analyses. The resulting design matrix also contained a standard 128s high-pass filter and motion estimates as covariates of no interest. Planned contrasts were then computed to create a contrast image of the main effect of each of the five conditions. These contrast images were then taken to a second-level factorial group analysis, including the same five conditions but different contrasts.

Effect(s) tested

We used a factorial design with the factors "subject" and "condition". There were 5 conditions: two for voice sounds (speech, nonverbal) and three for non-voice sounds (animal, natural, and artificial sounds). We tested the main effects of each of the five conditions on the subject-level. At the group-level we tested the following conditions against each other: voice, non-voice, speech, and nonverbal resulting in five t-contrasts and one F-contrast:
- voice > non-voice
- speech > non-voice
- nonverbal > non-voice
- speech > nonverbal
- nonverbal > speech
- F contrast (all 5 conditions)

Specify type of analysis: ☐ Whole brain ☐ ROI-based ☒ Both

Anatomical location(s)

Whole brain analysis was used to analyse brain activity during the different conditions.
ROI-based analysis was only used to extract time-courses for the DCM.
VOIs were based on the group-level brain activation peaks found in the three specific contrasts. We used the [voice > non-voice] contrast to define VOIs that represent voice processing (bilateral aST and pST), the [speech > non-verbal] contrast defined VOIs for speech processing (left mST and pSTS, right mST), and a F-contrast across all five conditions (i.e. higher activity in all conditions compared to baseline) defined areas in the HG as a general sound processing VOI in bilateral low-level AC. Thus, we defined five peak locations in the left hemisphere (HG, aST, mST, pST, pSTS) and four peak locations in the right hemisphere (HG, aST, mST, pST). Around each location, voxels within a sphere of a 3mm radius were selected. Peak locations for VOI definition were ensured to be >12mm apart from each other based on a Euclidian distance measure to ensure non-overlapping voxels in the VOIs and to take into account the smoothing kernel (6mm) for functional brain data. Only voxels that showed significant activation ($p<0.05$, uncorrected) by the presentation of all sounds within each participant were included, and voxel selection thus was adapted to individual activation profiles in each participant.

Statistic type for inference
(See Eklund et al. 2016)

Contrast images were thresholded at $p<0.05$ including a voxel-wise FWE correction.

Correction

Voxel-wise FWE correction.

Models & analysis

n/a	Involved in the study
☐	☒ Functional and/or effective connectivity
☒	☐ Graph analysis
☒	☐ Multivariate modeling or predictive analysis

Functional and/or effective connectivity

To examine effective functional connectivity, we used Dynamic causal modelling (DCM). From each VOI, the first principal component was extracted for the DCM analysis and adjusted for the F-contrast modelling the sound, voice, and speech regressors. Time series data from VOIs associated with the above regressors were summarized using the SPM12 eigenvariate toolbox.

For each participant and brain hemisphere, we first created a full connectivity model ("full model") with bidirectional connections between neighboring VOIs in each hemisphere (A matrix). Driving input (C matrix) to each node was specified by the experimental condition that elicited the original activity: The "all sounds" regressor provided input to HG, the "voice sound" regressor provided input to aST and pST, while the "speech sound" regressor provided input to mST (and additionally to left pSTS). The driving inputs were mean-centered, causing the parameters of the A matrix to represent the mean connection strengths across conditions. Finally, the modulation of intrinsic and extrinsic connections by experimental conditions (B matrix) followed the activation profile of the VOIs: Intrinsic connections in nodes were modulated by the activation profile of the node (e.g. intrinsic HG connectivity is modulated by "all sound" trials), connections from ST/STS regions to and from HG were set to be modulated by the ST/STS activation profile (e.g. HG-aST connections could only be modulated by the "voice sound" trials), and forward connections from ST/STS regions to other ST/STS region were set to be modulated by the activation profile of the origin of the connections (e.g. connections originating from mST could only be modulated by "speech sound" trials). We estimated these full DCM models for each participant using Bayesian model inversion.

To estimate group-level parameters in these left and right AC networks, we conducted a second-level

Parametric Empirical Bayes (PEB) analysis, separately for the left and right AC networks. The PEB analysis included a hierarchical model of the connectivity parameters, including connectivity parameters from all participants at the first-level as well as a GLM modelling at the second-level, and the estimation procedure used a variational scheme. After estimating the parameters of the full PEB model, we subsequently pruned away parameters using a Bayesian Model Reduction (BMR) approach performed on the A matrix and B matrix parameters. The BMR performs an automatic (greedy) search over the model space to optimize model evidence. The model evidence takes into account both model accuracy (how well the model fits the data) and model complexity (the difference between model parameters and their prior values). The BMR procedure was accomplished in an iterative process. Finally, we applied Bayesian Model Averaging (BMA) across all models searched by the BMR, and averaging was performed as a weighted average of parameters across models according to the posterior probabilities of the models. Significant network parameters were determined with a posterior probability of $p>0.99$.
